# Robotic Segmentectomy in a Patient with a Displaced Left Upper Division Bronchus and Fused Fissure

**DOI:** 10.70352/scrj.cr.25-0039

**Published:** 2025-03-25

**Authors:** Hironobu Wada, Ryo Karita, Yuki Hirai, Yuki Onozato, Toshiko Kamata, Hajime Tamura, Takashi Anayama, Ichiro Yoshino, Shigetoshi Yoshida

**Affiliations:** 1 Department of Thoracic Surgery, International University of Health and Welfare School of Medicine, Narita, Chiba, Japan; 2 Department of Thoracic Surgery, International University of Health and Welfare Narita Hospital, Narita, Chiba, Japan

**Keywords:** robotic-assisted thoracoscopic surgery, displaced bronchus, fused fissure, segmentectomy

## Abstract

**INTRODUCTION:**

In thoracic surgery, anatomical anomalies and a fused fissure can cause inaccurate intraoperative recognition of anatomy and lead to accidental injury of pulmonary vessels and bronchi that should be preserved. A displaced left upper division bronchus (B^1+2+3^), also known as a left eparterial bronchus, is a rare anomaly that can present in combination with abnormal pulmonary arteries positioning and lobulation. Herein, we report a case of lung cancer in S^1+2^ of the left fused lung that was successfully resected by robotic left upper division segmentectomy following a detailed preoperative simulation using 3-dimensional computed tomography.

**CASE PRESENTATION:**

A female octogenarian presented for the treatment of simultaneous bilateral lung cancer. Three months after surgery for right lung cancer, a surgery for left lung cancer was performed. Preoperative computed tomography identified several broncho-arterial anomalies and a completely fused fissure, including a displaced left upper division bronchus and a pulmonary artery running anteriorly to the left main bronchus, similar to those in the right lung. Robotic left upper division segmentectomy with lymph node dissection was performed using a “hilum first, fissure last” approach with fine dissection of the hilar structures and minimal bleeding. The postoperative course was uneventful.

**CONCLUSIONS:**

Preoperative simulation and robotic-assisted thoracoscopic surgery enabled the safe and precise anatomical pulmonary segmentectomy for a patient with lung cancer, despite several bronchial and arterial anomalies, including a displaced left upper division bronchus.

## Abbreviations

CT
: computed tomography
FDG
: ^18^F-fluorodeoxyglucose
FEV1
: forced expiratory volume in one second
LMB
: left main bronchus
RATS
: robot-assisted thoracoscopic surgery
UICC
: Union for International Cancer Control
ULB
: upper lobe bronchus
VATS
: video-assisted thoracoscopic surgery
3DCT
: 3-dimensional computed tomography

## INTRODUCTION

Preoperative simulation using 3-dimensional computed tomography (3DCT) is essential for thoracic surgeons to perform safe and precise anatomical lung resections, especially when patients have anatomical anomalies and incomplete lobulation of the lungs. A displaced left upper division bronchus (B^1+2+3^) is a rare anomaly, also known as a left eparterial bronchus, that can involve the left pulmonary artery running anteriorly to the left main bronchus (LMB) and cause abnormal lobulation. We report a case of lung cancer in the left upper lobe with a displaced left upper division bronchus, which was successfully resected by robotic left upper division segmentectomy following a detailed preoperative simulation using 3DCT.

## CASE PRESENTATION

**Fig. 1:**
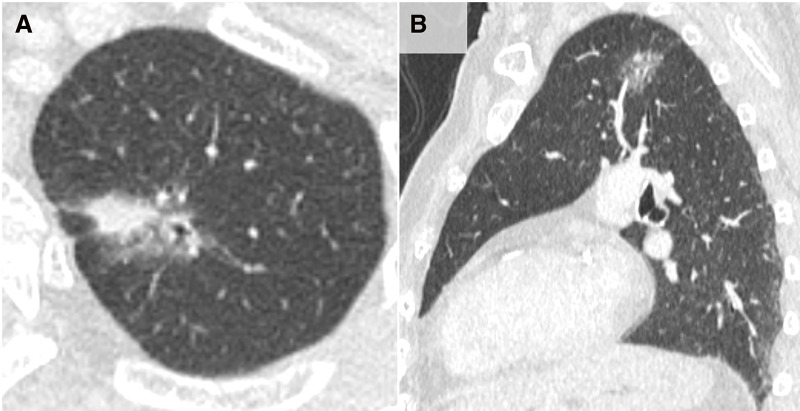
Preoperative computed tomography images. Preoperative computed tomography shows a part-solid ground-glass nodule in the left S^1+2^a involving V^1+2^b (**A**). The interlobar fissure is not visible (**B**).

A female octogenarian was referred to our department for the treatment of bilateral simultaneous double lung cancer. Video-assisted right S^6^ segmentectomy with lymph nodal dissection had been performed 3 months prior. The pathology revealed pT2aN0M0, IB (Union for International Cancer Control [UICC] 8th edition) adenocarcinoma. After recovering from the previous surgery, surgery for the left lung cancer was performed. The medical history included a myocardial infarction treated with percutaneous coronary intervention, which required continuous aspirin therapy. In addition, the patient had hypertension and type 2 diabetes mellitus that required medication. The patient was a never-smoker with a height and weight of 142.2 cm and 42.0 kg, respectively. Spirometry immediately before the left lung cancer surgery showed a forced vital capacity of 1.49 L (88.1% of predicted) and forced expiratory volume in 1 s (FEV_1_) of 1.01 L (80.8% of predicted). Chest radiography showed no abnormal shadows in the left lung field. Chest computed tomography (CT) showed a 2.3 cm part-solid ground-glass nodule with a solid diameter of 1.0 cm in S^1+2^ a near the intersegmental border with S^3^c (**Fig. 1**). Interlobar lines between the left upper and lower lobes were not confirmed. An abnormal accumulation of ^18^F-fluorodeoxyglucose (FDG) was showed by FDG positron-emission tomography/CT at the ground-glass nodule, with a standardized uptake value of 2.3, which was otherwise normal. The patient was diagnosed with c-T1aN0M0, IA1 (UICC 8th edition) lung cancer in the left upper lobe.

**Fig. 2:**
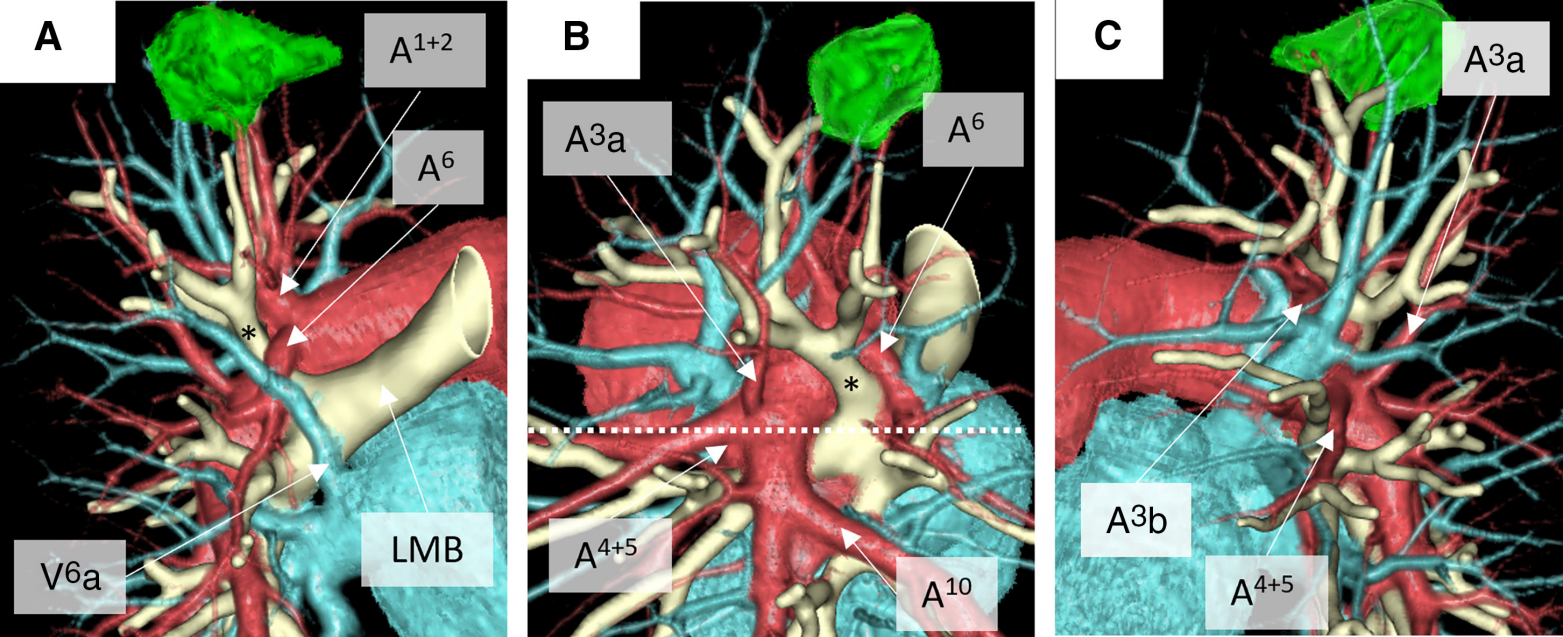
Preoperative 3-dimensional computed tomography reconstruction. Clockwise from the posterior (**A**) to the anterior (**C**) view. A left eparterial bronchus (*), or displaced left upper division bronchus (B^1+2+3^), with the pulmonary artery running anteriorly to the LMB. (**B**) A white dotted line shows the level where the left pulmonary artery crosses the LMB. A left eparterial bronchus originates from the LMB above the white dotted line. LMB, left main bronchus

**Fig. 3:**
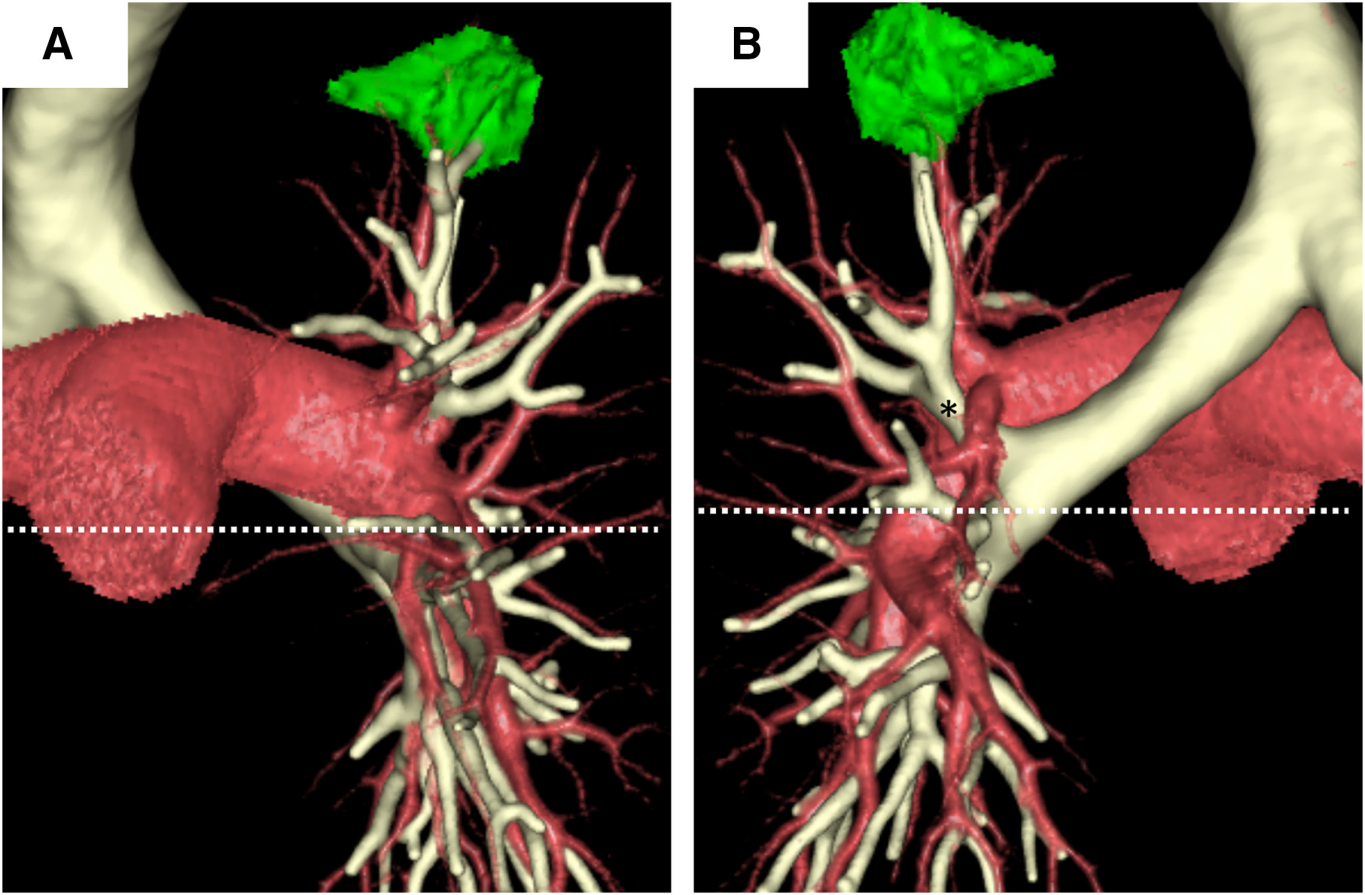
Eparterial bronchus in the left lung. Three-dimensional reconstruction of the pulmonary artery and bronchus from the (**A**) front and (**B**) back. A left eparterial bronchus (*) originates from the LMB superior to where the left pulmonary artery crosses the left main bronchus. The left pulmonary artery runs anteriorly to the LMB. A white dotted line indicates the level where the left pulmonary artery crosses the LMB. LMB, left main bronchus

**Fig. 4:**
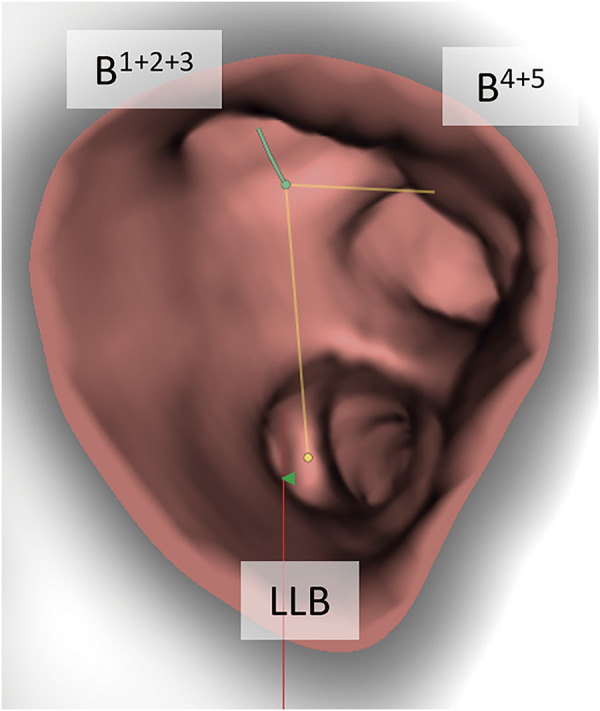
Three-dimensional reconstruction of the bronchial lumen. The orifices of 3 branches, including B^1+2+3^, B^4+5^, and the LLB, appear at the same level. LLB, left lower bronchus

Preoperative 3DCT identified several pulmonary artery and bronchial anomalies (**Fig. 2**). The left pulmonary artery ran anteriorly to the LMB, similar to that in the right lung (**Fig. 3**). The upper division bronchus, which was identified as a displaced bronchus, ascended between the descending trunk of the left pulmonary artery and A^6^, which arose at the level of A^1+2^. A^3^a branched at the level of A^4+5^ in the descending trunk of the left pulmonary artery. A 3-dimensional reconstruction of the bronchial lumen presented 3 equal branches of the left upper division bronchus, lingular bronchus, and the left lower lobe bronchus (**Fig. 4**). No pulmonary vein anomalies or abdominal organ anomalies were observed. Based on sufficient margin distance and adequate predicted postoperative FEV_1_, a robotic left upper division segmentectomy with lymph nodal dissection was planned.

**Fig. 5:**
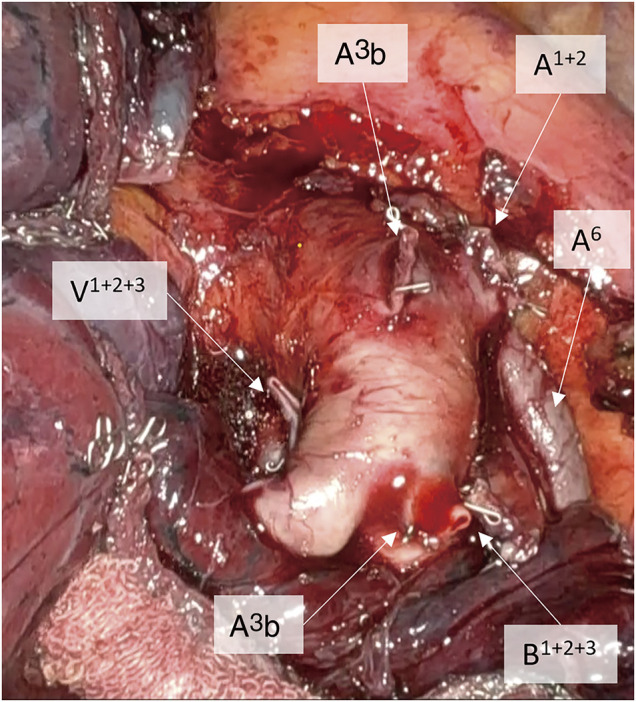
Intraoperative findings after left upper division segmentectomy. Transected branches of the pulmonary artery are indicated. The pulmonary artery runs just behind the stump of the pulmonary vein.

The Da Vinci Xi system (Intuitive Surgical, Sunnyvale, CA, USA) was used, and 4 ports were placed equally in the 7th intercostal space. The most ventral port was 12 mm in diameter, while the others were 8 mm. A 30° oblique angle camera was placed using the second arm. An assist port was located in the 10th intercostal space posteriorly. CO_2_ was insufflated into the left thoracic cavity. The interlobar fissure between the left upper and lower lobes was almost completely fused. First, A^1+2^ and A^6^, LMB, and V^6^a from top to bottom were identified behind the hilum. After flipping the lung, V^1–3^ was identified and transected. Then, the main pulmonary artery branching A^3^b in the central part was confirmed behind V^1–3^, where the left upper lobe bronchus (ULB) usually ascends. After transecting A^3^b, A^1+2^ ascending behind A^3^b was observed from the front. Next, behind the hilum, A^1+2^ was secured from the back and transected, avoiding injury to A^6^. Returning to the front again, A^3^a was identified near A^4+5^ and transected. The left upper division bronchus was identified behind the pulmonary artery and transected from the back. The intersegmental plane was divided from front to back using robotic staplers connected to the first arm, while moving the left lung and administering intravenous indocyanine green with near-infrared thoracoscopic imaging (Firefly Imaging System, Intuitive Surgical, Sunnyvale, CA, USA). The thoracoscopic findings after the left upper division segmentectomy are shown in **Fig. 5**. Superior mediastinum lymph nodes were sampled. Operative time was 245 min, and total blood loss was 10 g. The postoperative course was uneventful, without air leakage. The chest drainage tube was removed the next day, and the patient was discharged to home 7 days after surgery. Pathological analysis revealed pT1bN0M0, IA2 (UICC 8th edition) adenocarcinoma.

## DISCUSSION

The application of various imaging analysis software has enabled thoracic surgeons to understand the 3-dimensional anatomy of the lungs, and preoperative simulation using 3DCT has been useful not only for successful lung resection but also for surgical education.^1)^ Our institution uses 3DCT reconstruction for preoperative simulation in most cases, which was especially essential in this case and allowed us to recognize the branching pattern of the pulmonary artery and bronchus preoperatively, resulting in successful anatomical segmentectomy without accidental injury to the pulmonary arteries and bronchi.

A displaced bronchus is a bronchus that branches off in an abnormal position without a normal bronchus ventilating the corresponding parenchyma.^2)^ Its pathogenesis remains unknown; however, branching to the lobar and segmental bronchi occurs by 30–37 and 36–41 days of gestation, respectively, and a displaced bronchus is hypothesized to occur after 32 days, as bronchi elongate and branch further.^2–4)^ Since the formation of the airway bronchial tree may serve as a template for the blood vessel vascular tree,^4)^ displaced bronchi are presumed to be the cause of the abnormal positioning of pulmonary arteries. Various types of displaced bronchi have been described, and many are easily overlooked on preoperative chest CT, which increases the risk of intraoperative complications.^5)^ The most reliable approach for the correct diagnosis of displaced bronchi in the left upper lobe is to examine the relationship of the ULB to the ipsilateral pulmonary artery. The LMB usually runs beneath the left pulmonary artery, and the left ULB originates below the point where the left pulmonary artery crosses the LMB; thus, the normal left ULB is considered hyparterial.^2)^ In contrast, most displaced left upper division bronchi are eparterial. A left eparterial bronchus refers to any bronchus directed toward the left upper lobe that arises from the LMB superior to where the left pulmonary artery crosses the LMB. The exact frequency of a left eparterial bronchus is uncertain but is estimated to be <1%.^3,5)^ A left eparterial bronchus is also frequently associated with other tracheobronchial anomalies and accessory fissures.^5)^ This case was consistent with a left eparterial bronchus, and no accessory fissures or other tracheobronchial anomalies were observed.

Robot-assisted thoracoscopic surgery (RATS) has been widely accepted as a minimally invasive approach to lung cancer treatment because of its various advantages, with short- and long-term outcomes that are comparable to those of video-assisted thoracoscopic surgery (VATS).^6,7)^ A recently published meta-analysis comparing the outcomes of RATS versus VATS in the treatment of lung cancer, which included only randomized controlled trials or prospective cohort studies, reported that RATS had a clear advantage in lymphadenectomy and control of hemorrhage.^6)^ This is likely due to the advantages of flexible equipment and a 3-dimensional magnified vision, which help reveal complex anatomy accurately, leading to precise manipulation and better control of bleeding.^6)^ Hao et al.^8)^ found that robot-assisted anatomical lung resection is safe and effective, even in patients with incomplete lobulation.

Their technique was “fissure first, hilum last”, which shortened operative time, even with some lung parenchymal injury. During our operation, owing to several anomalies and the completely fused lung, we focused on ensuring that the pulmonary hilum was fully dissected both anteriorly and posteriorly to avoid misjudging the anatomic structures. After transecting all affected pulmonary vessels and bronchus, the intersegmental plane was divided as the final step. Even though creating operating fields multiple times during RATS prolongs the operative time, this “hilum first, fissure last” approach following careful dissection of the hilar structures was necessary to avoid misinterpretation of the anatomy and success anatomical segmentectomy. Although the same approach is possible with VATS, we believe that robotic surgery was beneficial in fully exposing the hilar structures with only minimal bleeding in this case, which involved a complicated anatomy. In addition, the fluorescent visualization equipped in the robotic system was useful in such a fused fissure case.

## CONCLUSIONS

Preoperative simulation with 3DCT reconstruction and the use of a robotic platform aided us in performing a safe and precise anatomical pulmonary segmentectomy, even in a patient with several arterial and bronchial anomalies, including a displaced left upper division bronchus.

## ACKNOWLEDGMENTS

We would like to thank Editage (www.editage.com) for English language editing. We would also like to acknowledge Mr. Daisuke Tomoshige and Ms. Mamiko Igarashi for their support in reconstructing the 3DCT images.

## DECLARATIONS

### Funding

This research did not receive any specific grants from funding agencies in the public, commercial, or not-for-profit sectors.

### Authors’ contributions

Article writing: HW.

Data collection: HW.

Clinical practice: HW, RK, YH, YO, TK, HT, TA, IY, and SY.

Proofing: HW, RK, YH, YO, TK, HT, TA, IY, and SY.

All authors agree to take responsibility for all aspects of the study.

All authors have read and approved the manuscript.

### Availability of data and materials

There is no dataset supporting the conclusions of this article.

### Ethics approval and consent to participate

The need for ethics approval was waived owing to the nature of a case report.

### Consent for publication

Written consent was obtained from the patient for publication.

### Competing interests

The authors declare that they have no competing interests.

## References

[1] Hamanaka K Miura K Eguchi T Harnessing 3D-CT simulation and planning for enhanced precision surgery: A review of applications and advancements in lung cancer treatment. Cancers (Basel) 2023; 15: 5400. 38001660 10.3390/cancers15225400 PMC10670431

[2] Chassagnon G Morel B Carpentier E Tracheobronchial branching abnormalities: lobe-based classification scheme. Radiographics 2016; 36: 358–73. 26824513 10.1148/rg.2016150115

[3] Ghaye B Szapiro D Fanchamps JM Congenital bronchial abnormalities revisited. Radiographics 2001; 21: 105–19. 11158647 10.1148/radiographics.21.1.g01ja06105

[4] Schittny JC. Development of the lung. Cell Tissue Res 2017; 367: 427–44. 28144783 10.1007/s00441-016-2545-0 PMC5320013

[5] Oshiro Y Murayama S Ohta M CT findings of a displaced left upper division bronchus in adults: its importance for performing safe left pulmonary surgery. Eur J Radiol 2013; 82: 1347–52. 23480963 10.1016/j.ejrad.2013.02.003

[6] Huang S Huang X Huang Z Comparison of robot-assisted thoracic surgery versus video-assisted thoracic surgery in the treatment of lung cancer: a systematic review and meta-analysis of prospective studies. Front Oncol 2023; 13: 1271709. 38023124 10.3389/fonc.2023.1271709 PMC10646752

[7] Tasoudis PT Diehl JN Merlo A Long-term outcomes of robotic versus video-assisted pulmonary lobectomy for non-small cell lung cancer: systematic review and meta-​analysis of reconstructed patient data. J Thorac Dis 2023; 15: 5700–13. 37969301 10.21037/jtd-23-582 PMC10636447

[8] Hao X Jun W Xiaoyan C Robot-assisted thoracic surgery for lung cancer patients with incomplete fissure. Surg Endosc 2022; 36: 8290–7. 35552813 10.1007/s00464-022-09283-x

